# Prostate involvement during sexually transmitted infections as measured by prostate-specific antigen concentration

**DOI:** 10.1038/bjc.2011.271

**Published:** 2011-07-26

**Authors:** S Sutcliffe, R L Nevin, R Pakpahan, D J Elliott, S R Cole, A M De Marzo, C A Gaydos, W B Isaacs, W G Nelson, L J Sokoll, J M Zenilman, S B Cersovsky, E A Platz

**Affiliations:** 1 Division of Public Health Sciences and the Alvin J. Siteman Cancer Center, Department of Surgery, Washington University School of Medicine, 660 South Euclid Avenue, Box 8100, Room 5026, St Louis, MO 63110, USA; 2 Department of Preventive Medicine, Bayne-Jones Army Community Hospital, 1585 3rd Street, Folk Port, LA 71459, USA; 3 Division of Public Health Sciences, Department of Surgery, Washington University School of Medicine, 660 South Euclid Avenue, Box 8100, Room 5019, St Louis, MO 63110, USA; 4 Department of Pathology, Johns Hopkins Medical Institutions, 600 North Wolfe Street, Meyer B-194, Baltimore, MD 21287, USA; 5 Department of Epidemiology, Gillings School of Global Public Health, University of North Carolina at Chapel Hill, McGavran-Greenberg Hall, Campus Box 7435, Chapel Hill, NC 27599, USA; 6 Departments of Pathology and Oncology, James Buchanan Brady Urological Institute, and the Sidney Kimmel Comprehensive Cancer Center, Johns Hopkins Medical Institutions, CRB-1 Room 151, 1650 Orleans Street, Baltimore, MD 21205, USA; 7 Division of Infectious Diseases, Department of Medicine, Johns Hopkins Medical Institutions, 855 North Wolfe Street, 530 Rangos Building, Baltimore, MD 21205, USA; 8 James Buchanan Brady Urological Institute and the Sidney Kimmel Comprehensive Cancer Center, Johns Hopkins Medical Institutions, 115 Marburg Building, 600 North Wolfe Street, Baltimore, MD 21205, USA; 9 Departments of Oncology, Pathology, and Pharmacology, James Buchanan Brady Urological Institute, and the Sidney Kimmel Comprehensive Cancer Center, Johns Hopkins Medical Institutions, 401 North Broadway Street, The Weinberg Building, Suite 1100, Baltimore, MD 21231, USA; 10 Department of Pathology, James Buchanan Brady Urological Institute, and the Sidney Kimmel Comprehensive Cancer Center, Johns Hopkins Medical Institutions, 600 North Wolfe Street, Meyer B-125, Baltimore, MD 21287, USA; 11 Division of Infectious Diseases, Department of Medicine, Johns Hopkins Medical Institutions, 4940 Eastern Avenue, Baltimore, MD 21224, USA; 12 US Army Institute of Public Health, US Army Public Health Command (Provisional), 5158 Blackhawk Road, Aberdeen Proving Ground, Aberdeen, MD 21010, USA; 13 Department of Epidemiology, Johns Hopkins Bloomberg School of Public Health, and the James Buchanan Brady Urological Institute and Sidney Kimmel Comprehensive Cancer Center, Johns Hopkins Medical Institutions, 615 North Wolfe Street, Room E6132, Baltimore, MD 21205, USA

**Keywords:** sexually transmitted infections, chlamydia, gonorrhoea, non-chlamydial, non-gonococcal urethritis, prostate-specific antigen, prostate cancer

## Abstract

**Background:**

We investigated prostate involvement during sexually transmitted infections by measuring serum prostate-specific antigen (PSA) as a marker of prostate infection, inflammation, and/or cell damage in young, male US military members.

**Methods:**

We measured PSA before and during infection for 299 chlamydia, 112 gonorrhoea, and 59 non-chlamydial, non-gonococcal urethritis (NCNGU) cases, and 256 controls.

**Results:**

Chlamydia and gonorrhoea, but not NCNGU, cases were more likely to have a large rise (⩾40%) in PSA than controls (33.6%, 19.1%, and 8.2% *vs* 8.8%, *P*<0.0001, 0.021, and 0.92, respectively).

**Conclusion:**

Chlamydia and gonorrhoea may infect the prostate of some infected men.

Despite ongoing interest in the role of exudative sexually transmitted infections (STIs; i.e., those that cause an inflammatory discharge) in prostate carcinogenesis (Sutcliffe, 2010), few studies have examined the likelihood of prostate involvement during infection, particularly in the current antibiotic era (Sutcliffe and Platz, 2007). We previously investigated this question by measuring serum prostate-specific antigen (PSA) as a marker of prostate infection, inflammation, and/or cell damage in a small study of young, African-American STI patients (Sutcliffe *et al*, 2006). We used PSA as a marker because it rises in men with acute bacterial prostatitis and asymptomatic histologic prostate inflammation (Sindhwani and Wilson, 2005). In our previous study, we found that men with exudative STIs were more likely to have a large rise (⩾40%) in PSA during infection than in controls, suggesting that prostate infection occurred in some infected men. To investigate the reproducibility of these findings and to determine which STIs predict PSA elevation, we have now conducted a considerably larger study among US military members with stored serum in the Department of Defense serum repository (DoDSR).

## Subjects and methods

### Study population and design

The DoDSR contains serum remaining from human immunodeficiency virus type 1 (HIV-1) screening during pre-induction, at routine periodic intervals (every 2–5 years), before and after major overseas deployments, for clinical indications, and as part of standard clinical STI work-up. Specimens are linked to demographic information, service-related activity, and reportable (e.g., genital, chlamydial infection; gonorrhoea; and non-chlamydial, non-gonococcal urethritis (NCNGU)) and non-reportable medical diagnoses (Rubertone and Brundage, 2002; Silverberg *et al*, 2003).

Men eligible for the present study were those who were <25 years of age as of 1995; HIV-1 negative; on continuous active duty from 1995 to 2006; and had several archived specimens in the DoDSR (*n*=75 387). We defined STI cases as men with a laboratory-confirmed diagnosis of chlamydia (ICD-9-CM code 099.41), gonorrhoea (098), or NCNGU (099.40) in 2001–2003. Diagnoses were confirmed according to military guidelines; NCNGU required specific exclusion of chlamydia and gonorrhoea (Army Medical Surveillance Activity, 1998). We defined controls as men with no STI or infectious mononucleosis diagnoses in their medical record up to 2006. Exclusion of mononucleosis diagnoses was needed for a separate investigation. Controls were frequency matched to the entire case group by race.

For each case, we selected two specimens from the DoDSR, one collected ±7 days of the case diagnosis (acute), and the first specimen collected >3 weeks before their acute specimen to account for the maximum typical incubation period of chlamydia and gonorrhoea (Hook and Handsfield, 2008; Stamm, 2008; pre-acute, range: 22 days–4 years before diagnosis). If a case had ⩾2 diagnoses, only one was selected. Diagnoses without a specimen collected ±7 days were excluded. These criteria resulted in a sample size of 299 chlamydia, 112 gonorrhoea, and 59 NCNGU cases. Two specimens were also selected for each control, one collected from 2001 to 2003 (‘acute’) and the first specimen collected >3 weeks before their acute specimen (‘pre-acute’). If ⩾2 ‘acute’ specimens were available, one was randomly selected. Of the 68 584 eligible controls, we selected 256 based on power considerations and available resources.

This study was approved by the Walter Reed Army Institute of Research and Johns Hopkins. All data/specimens were anonymised before release from the DoDSR.

### PSA measurement

As participants were younger than the age range for routine prostate cancer screening, PSA was not available in their medical records. Therefore, we measured total PSA for all participants using the Access Hybritech assay (Beckman Coulter, Brea, CA, USA). Specimens from the same individual were tested adjacent to one another in random within-person order. We determined assay reproducibility by testing 25 blinded quality control pairs from the DoDSR (coefficient of variation=12.4%, and 6.9% after excluding one discrepant pair). Total PSA has been found to be relatively stable for 2–20 years at −20 °C (Woodrum and York, 1998; Ulmert *et al*, 2006), close to the storage temperature in the DoDSR (−30 °C).

### Statistical analysis

We initially explored PSA change between the pre-acute and acute specimens by comparing mean pre-acute and acute PSA for cases and controls. Values were adjusted for race to account for frequency matching. We further explored PSA change by comparing race-adjusted categories of absolute and relative percent change. As in our previous analysis (Sutcliffe *et al*, 2006), case and control distributions diverged at a 40–49% PSA rise; therefore, we used a ⩾40% change to define a large rise.

## Results

We identified 299 cases of chlamydia, 112 of gonorrhoea, and 59 of NCNGU, and selected 256 controls for comparison. Compared with controls, cases were slightly younger; gonorrhoea cases were more likely to be African-American; and all cases were more likely to be unmarried, enlisted, and to have had their blood drawn for clinical indications (Table 1). Cases also had a greater number of blood draws, and a correspondingly shorter time between draws, particularly between their pre-acute and acute specimens.

In general, STI cases had a higher mean pre-acute PSA than controls (*P*=0.0023; Table 2). Comparing pre-acute and acute specimens, chlamydia cases had a significantly greater mean change in PSA between specimens than controls, and were more likely to have a large rise in PSA at the time of their acute specimen, as defined by both absolute and relative change. For gonorrhoea, although cases did not have a significantly greater mean change in PSA than controls, they were more likely to have both a large absolute and relative rise. The magnitude of this difference was, however, smaller than for chlamydia (*P*=0.0005). Non-chlamydial, non-gonococcal urethritis cases were no more likely to have a large PSA rise than controls. No changes were observed after adjustment for age, calendar year of the pre-acute specimen, time between specimens, and pre-acute PSA. Only one of the four cases with recorded prostate symptoms (ICD-9-CM code 601) at the time of their STI diagnosis had a large PSA rise.

As men were not necessarily tested for STIs at the time of their pre-acute specimen, we repeated the analyses restricted to men with ⩾1 year between their pre-acute and acute specimens to remove men more likely to have been infected with their subsequently diagnosed acute STI at the time of their pre-acute specimen; in general, similar results were obtained. We also investigated the influence of additional diagnosed or undiagnosed STIs on the results by excluding: (1) men with additional infectious or genitourinary diagnoses immediately before their pre-acute specimen or between specimens; (2) cases with clinical or other suspicion of HIV/STIs as their reason for blood draw for their pre-acute specimen, and controls with these reasons for either specimen; (3) men with small breaks (<60 days) in their active duty status or deployed between specimens; and (4) higher rank officers who may have greater access to non-military health care. All sensitivity analyses yielded similar results as the main analyses (data not shown).

## Discussion

In this large study of US military members, men with exudative STIs were more likely to have a large PSA rise during infection than controls, similar to findings from our previous smaller study of STI patients (Sutcliffe *et al*, 2006). This rise was observed for chlamydia and gonorrhoea, but not for NCNGU. Although cases were also more likely to have higher pre-acute PSA, this difference should not have influenced inferences for PSA change because similar findings were observed for absolute and relative change, only the latter of which varies depending on the pre-acute value.

While all STIs had the potential for prostate involvement (Sutcliffe and Platz, 2007, 2008), chlamydia cases were most likely to have a large PSA rise, followed by gonorrhoea cases, whereas NCNGU cases were no more likely to have a rise than controls. One possible reason for these differences may be likelihood of symptoms as a possible marker of duration of infection. We previously hypothesised that men with asymptomatic infections/non-specific symptoms might be more likely to have prostate infection because of their likely lesser awareness of their STI and consequent delay seeking treatment. This delay might provide pathogens with greater opportunity to ascend to and infect the prostate, which we believe is a likely necessary first step for prostate carcinogenesis (Sutcliffe *et al*, 2006). This hypothesis is consistent with differences in the likelihood of symptoms for each STI. Approximately half of chlamydial infections are asymptomatic in men (Zimmerman *et al*, 1990), a large proportion of which are ultimately diagnosed in the military because of routine female screening, contact tracing, and self-referral for risky behaviours; 1–49% of gonorrhoeal infections present without symptoms (Pedersen and Harrah, 1970; Turner *et al*, 2002); and a likely even smaller proportion of diagnosed NCNGU infections present without symptoms because NCNGU is typically only investigated in men with urethral symptoms. An alternative explanation for null NCNGU findings, but not for differences between chlamydia and gonorrhoea, is that some NCNGU cases did not have an infectious aetiology.

Although we used PSA as a marker of prostate infection/inflammation/cell damage, PSA may also possibly rise as a result of a more generalised response to infection at other non-prostate sites (e.g., urethra), recent ejaculation (within 1 day), or digital rectal examination (DRE; Tchetgen and Oesterling, 1997). We believe these possibilities are less likely because of null results for NCNGU, an STI defined by urethral inflammation; unchanged results after adjustment for sexual activity in our previous study (Sutcliffe *et al*, 2006); and the extreme rarity with which DREs are performed during routine STI work-up, especially in the military.

In summary, young men with chlamydia and gonorrhea were more likely to have a large PSA rise during infection than controls. Future studies should investigate the specificity of this rise to prostate infection, as well as the long-term effects of infections on PSA and, by possible extension, the prostate environment.

## Figures and Tables

**Table 1:** Demographic characteristics of 470 young, male sexually transmitted infection (STI) cases and 256 controls, US military 2001–2003^a^

	**Controls (*n*=256)**	**Chlamydia cases (*n*=299)**	**Gonorrhoea cases (*n*=112)**	**NCNGU cases (*n*=59)**	***P*-value^b^**
Mean age (years)^c^	29.9	29.2	29.0	29.1	<0.0001 (<0.0001)
					
*Race/ethnicity (%)* d					
African-American	55.1	54.2	79.5	52.5	
Caucasian-American	36.3	36.1	16.1	35.6	0.0007 (0.27)
Other	8.6	9.7	4.4	11.9	
					
*Marital status (%)* c					
Married	79.3	60.7	63.5	73.4	<0.0001 (<0.0001)
Other	20.7	39.3	36.5	26.6	
					
*Military grade (%)* c					
Enlisted	91.1	96.2	97.3	99.0	0.011 (0.0012)
Officer	8.9	3.8	2.7	1.0	
					
*Reason for blood draw (%)* c					
Routine^e^	69.6	22.3	25.0	17.6	
Clinically indicated/part of an STI visit^f^	1.9	34.2	48.7	70.4	<0.0001 (<0.0001)
Other/unknown	28.5	43.5	26.4	12.0	
					
Mean number of blood draws for HIV-1 testing from 1 January 1995 to 31 December 2006	9.9	12.0	11.3	13.4	<0.0001 (<0.0001)
Mean time between pre-acute and acute specimens (months)	16.8	11.9	12.0	10.7	<0.0001 (<0.0001)

Abbreviations: HIV-1=human immunodeficiency virus type 1; NCNGU=non-chlamydial, non-gonococcal urethritis.

a Values for cases and controls were calculated by linear regression adjusting for race (African-American and non-African-American), except for values for the race variable.

b *P*-values were calculated by linear regression for continuous or binary variables, and by generalised logit regression for categorical variables. All models were adjusted for race (African-American and non-African-American). *P*-values reflect a test of independence across all groups. Values in parentheses compare all cases with controls.

c At the time of blood draw of the acute specimen.

d Cases were frequency matched to controls by race/ethnicity.

e Indicates blood drawn for routine and pre- and post-deployment HIV-1 tests, as well as HIV-1 tests performed as part of specialised physical examinations (e.g., for flight school).

f Indicates blood drawn for self or clinical suspicion of HIV-1 or STIs, as well as from hospitalised patients or those visiting emergency rooms for certain clinical indications. Blood draws are coded as ‘clinically indicated/part of an STI visit’ irrespective of the results of HIV-1 or STI testing.

**Table 2:** Pre-acute and acute serum total prostate-specific antigen (PSA) concentration for 470 young, male sexually transmitted infection cases and 256 controls, US military 2001–2003

	**Controls (*n*=256)**		**Chlamydia cases (*n*=299)**			**Gonorrhoea cases (*n*=112)**			**NCNGU cases (*n*=59)**		
*PSA (ng ml* ^*−1*^ *)*	*Pre-acute*	*Acute*	*Pre-acute*	*Acute*	P*-value*^a^	Pre-acute	*Acute*	P*-value*^a^	Pre-acute	*Acute*	P*-value*^a^
Geometric mean^b^	0.55	0.56	0.62	0.82	<0.0001	0.66	0.70	0.47	0.63	0.64	0.84
Mean^b^	0.64	0.65	0.86	1.11	0.0078	0.80	0.93	0.32	0.70	0.82	0.33
Range^c^	0.38–0.76	0.38–0.80	0.43–0.87	0.52–1.33		0.44–0.98	0.44–1.04		0.48–0.82	0.46–0.86	
											
*Distribution of absolute change in serum total PSA (%)* b											
⩽0.00 ng ml^−1^	52.1		36.2			45.1			48.8		
0.01–0.09 ng ml^−1^	24.7		19.8			31.1			30.6		
0.10–0.19 ng ml^−1^	15.1		9.0			4.0			10.5		
0.20–0.29 ng ml^−1^	2.8		7.4		<0.0001	6.1		0.0023	3.5		
0.30–0.39 ng ml^−1^	2.1		5.5			1.5			1.9		0.57
0.40–0.49 ng ml^−1^	1.6		2.4			1.5			0.1		
⩾0.50 ng ml^−1^	1.6		19.7			10.6			4.6		
							
*Distribution of relative percent change in serum total PSA (%)* b											
⩽0%	52.1		36.2			45.1			48.8		
0.1–9%	11.7		12.7			21.7			12.3		
10–19%	12.8		7.9			10.0			18.5		
20–29%	10.1		5.6		<0.0001	1.9		0.0044	11.8		0.38
30–39%	4.5		3.9			2.2			0.3		
40–49%	1.8		5.6			3.8			3.3		
⩾50%	7.0		28.0			15.3			5.0		
							
*Large relative rise in PSA (%)* b											
⩾40%	8.8		33.6		<0.0001	19.1		0.021	8.2		0.92

Abbreviation: NCNGU=non-chlamydial, non-gonococcal urethritis.

a *P*-values were calculated by linear regression with robust variance estimation for continuous variables, logistic regression for categorical variables and linear regression for binary variables. All models were adjusted for race (African-American and non-African-American).

b Values were calculated by linear regression adjusting for race (African-American and non-African-American).

c Not adjusted for race.
